# α-Hederin Arrests Cell Cycle at G2/M Checkpoint and Promotes Mitochondrial Apoptosis by Blocking Nuclear Factor-κB Signaling in Colon Cancer Cells

**DOI:** 10.1155/2018/2548378

**Published:** 2018-09-27

**Authors:** Dongdong Sun, Weixing Shen, Feng Zhang, Huisen Fan, Jiani Tan, Liu Li, Changliang Xu, Haibin Zhang, Ye Yang, Haibo Cheng

**Affiliations:** ^1^The First Clinical Medical College, Nanjing University of Chinese Medicine, Nanjing 210023, China; ^2^Key Laboratory of Famous Doctors' Proved Recipe Evaluation and Transformation under State Administration of Traditional Chinese Medicine, Jiangsu Provincial Laboratory of Proved Anticarcinoma Recipe Research and Industrialization Engineering, Jiangsu Collaborative Innovation Center of Traditional Chinese Medicine Prevention and Treatment of Tumor, Nanjing University of Chinese Medicine, Nanjing 210023, China; ^3^College of Pharmacy, Nanjing University of Chinese Medicine, Nanjing 210023, China; ^4^School of Medicine and Life Sciences, Nanjing University of Chinese Medicine, Nanjing 210023, China

## Abstract

Colon cancer represents the third most common malignancy worldwide. New drugs with high efficaciousness and safety for the treatment of colon cancer are urgently needed in clinical context. Here, we were aimed to evaluate the antitumor activity of the natural compound *α*-hederin in human colon cancer cells. We treated SW620 cells with interleukin-6 (IL-6)* in vitro *to mimic the paracrine inflammatory microenvironment of tumor cells. *α*-Hederin concentration dependently reduced the viability of IL-6-stimulated SW620 cells. *α*-Hederin increased the number of IL-6-stimulated SW620 cells at the G2/M phase and reduced the mRNA and protein expression of cyclin B1 and CDK1. Moreover, *α*-hederin induced apoptosis and loss of mitochondrial membrane potential in IL-6-stimulated SW620 cells. *α*-Hederin downregulated Bcl-2 expression, upregulated Bax expression, and promoted cytochrome c release from mitochondria into cytoplasm. Additionally, *α*-hederin elevated the levels of cleaved-caspase-9, cleaved-caspase-3, and cleaved-PARP, but had little effects on the levels of cleaved-caspase-8. Moreover, *α*-hederin prevented the nuclear translocation of nuclear factor-*κ*B (NF-*κ*B) and reduced the phosphorylation of I*κ*B*α* and IKK*α*, suggesting the blockade of NF-*κ*B signaling. NF-*κ*B inhibitor PDTC not only produced similar proapoptotic effects on IL-6-stimulated SW620 cells as *α*-hederin did, but also synergistically enhanced *α*-hederin's proapoptotic effects. Furthermore, *α*-hederin inhibited the phosphorylation of ERK in IL-6-stimulated SW620 cells, which was involved in *α*-hederin blockade of NF-*κ*B nuclear translocation. Altogether, *α*-hederin suppressed viability, induced G2/M cell cycle arrest, and stimulated mitochondrial and caspase-dependent apoptosis in colon cancer cells, which were associated with disruption of NF-*κ*B and ERK pathways, suggesting *α*-hederin as a promising candidate for intervention of colon cancer.

## 1. Introduction

Colon cancer represents the third most frequently diagnosed malignancy all over the world. Although the incidence has been decreased during the past decade, colon cancer still has considerably high morbidity compared with other cancers [1]. Despite the surgical resection as a primary curative management for the early stage of colon cancer, a growing number of patients are in the advanced stage at the time of diagnosis because of the lack of effective screening [2]. Chemotherapy still serves as a common therapeutic strategy for colon cancer. Unfortunately, many patients eventually relapse, and chemotherapy resistance occurs [3]. Thus, development of novel agents with high efficaciousness and safety against colon cancer is of extreme importance.

Apoptosis is a genetically controlled cell death, playing an important role in regulation of development, cell proliferation, and stress responses [4]. Tumorigenesis has long-termly been linked to alterations of apoptotic pathways [5]. The mechanisms underlying tumor chemotherapy resistance are also largely associated with the activation of antiapoptotic pathways and the sensitivity of tumor cells to drug-induced apoptosis is determined by the balance between the antiapoptotic and proapoptotic signals [5]. Mitochondria play a pivotal role in apoptosis. During apoptosis, some key events occur in mitochondria, including the loss of mitochondrial membrane potential (MMP), reduced ratio of Bcl-2/Bax, and release of cytochrome c (Cyt c). Particularly, MMP is a key parameter of mitochondrial apoptosis [6]. On the other hand, excessive activation of nuclear factor *κ*B (NF-*κ*B) has been observed in many solid tumors, especially in colon cancer [7]. NF-*κ*B with transcription activity facilitates the progression of colon cancer by regulating the expression of a range of apoptosis-related genes, including Bcl-2, Bcl-xl, Survivin, etc. NF-*κ*B also mediates survival mechanisms through upregulating antiapoptotic genes. Therefore, NF-*κ*B signaling is a causative factor in chemotherapy resistance [8].

Natural products provide promising candidate agents for cancer chemotherapy. *α*-Hederin is a monodesmosidic triterpenoid saponin isolated from the leaves of* Hedera helix*. Recent studies have demonstrated that *α*-hederin had multiple pharmacological effects such as anti-virus, anti-oxidation, anti-inflammation, etc. [9, 10]. In current study, we examined the effects of *α*-hederin on the fate of colon cancer cells, and to explore the underlying mechanisms focusing on the NF-*κ*B pathway. Because tumorigenesis and progression are commonly driven by inflammatory microenvironment, we treated colon cancer cells with interleukin 6 (IL-6, a well-known inflammatory cytokine) to mimic the inflammatory microenvironment* in vitro*.

## 2. Materials and Methods

### 2.1. Reagents and Antibodies

*α*-Hederin of purity over 99% was provided by Shanghai Yuanye Biotechnology Co., Ltd. (Shanghai, China). NF-*κ*B specific inhibitor pyrrolidine dithiocarbamate (PDTC), JAK2/STAT3 signaling specific inhibitor AG490, and ERK specific inhibitor U0126 were obtained from Selleck Chemicals (Houston, TX, USA). Dimethyl sulfoxide (DMSO) was used to dissolve the above compounds for experiments, and single treatment with DMSO was used as negative control. Recombinant human IL-6 cytokine was obtained from Solarbio Life Science (Beijing, China). The primary antibodies used for Western blot analyses against cyclin B1, CDK1, Bcl-2, Bax, Cyt c, cleaved-caspase-9, cleaved-caspase-3, cleaved-caspase-8, cleaved-PARP, NF-*κ*B(p65), p-I*κ*B*α*, I*κ*B*α*, p-IKK*α*, IKK*α*, p-ERK, ERK, COX IV, lamin B1, and GAPDH were purchased from Bioworld Technology, Inc. (MN, USA).

### 2.2. Cell Culture

Human colon cancer HCT116 and SW620 cells were provided by the Cell Bank of Chinese Academy of Sciences (Shanghai, China). Cells were cultured in RPMI-1640 medium incubated with 10% fetal bovine serum (FBS), 50 U/ml penicillin, and 50 *μ*g/ml streptomycin according to our previous methods [11]. Cells were grown at 37°C in a 5% CO_2_ incubator.

### 2.3. Cell Viability Assay

HCT116 or SW620 cells were seeded in 96-well plates and then treated with vehicle, IL-6, and/or *α*-hederin, or AG490 at different concentrations for 24 h. Cell Counting Kit-8 (Nanjing Enogene Biotechnology. Co., Ltd., Nanjing, China) was used to evaluate cell viability according to the protocol as we previously reported [11]. The spectrophotometric absorbance at 450 nm was determined using a SPECTRAmax™ microplate spectrophotometer (Molecular Devices, Sunnyvale, CA, USA). Cell viability was presented as the percentage of control values.

### 2.4. Cell Cycle Analysis by Flow Cytometry

SW620 cells were seeded in 6-well plates and then treated with vehicle, IL-6, and/or *α*-hederin at different concentrations for 24 h. Cells were fixed, and cell cycles were monitored using a cellular DNA flow cytometric analysis kit (Nanjing KeyGen Biotech Co., Ltd., Nanjing, China) in accordance with the protocol. Percentages of cells within the G0/G1, S, and G2/M phases were detected using flow cytometry (FACSCalibur; Becton, Dickinson and Company, Franklin Lakes, NJ, USA). Results were from triplicate experiments.

### 2.5. Hoechst 33258 Staining

SW620 cells were seeded in 6-well plates and then treated with vehicle, IL-6, and/or *α*-hederin, or PDTC at different concentrations for 24 h. Apoptosis was evaluated using a Hoechst 33258 staining kit provided by Beyotime Institute of Biotechnology (Haimen, China) according to the protocol. Morphology of apoptotic cells was observed using a fluorescence microscope (Nikon, Tokyo, Japan). The nucleus of apoptotic cells takes up the Hoechst reagent and exhibits a bright blue fluorescence. Results were from experiments in triplicate.

### 2.6. Flow Cytometry Analysis of MMP

SW620 cells were seeded in 6-well plates and then treated with vehicle, IL-6, and/or *α*-hederin at different concentrations for 24 h. MMP was determined using flow cytometry. Briefly, cells were incubated separately with 5 mM JC-1 dye provided by Beyotime Institute of Biotechnology (Haimen, China) at 37°C for 30 min followed by centrifugation (300 g, 5 min) and suspension in phosphate buffer solution. The fluorescence-labeled cells were monitored by flow cytometry at the excitation and emission wavelengths of 530 nm and 590 nm, respectively, using FACS Calibur Flow Cytometry System (BD Biosciences, Franklin Lakes, NJ, USA). Results were from experiments in triplicate.

### 2.7. Immunofluorescence Staining

SW620 cells were seeded in 6-well plates, and then treated with vehicle, IL-6, and/or *α*-hederin at different concentrations for 24 h. Then, cells were incubated with the primary antibody against NF-*κ*B(p65) and fluorescence-conjugated secondary antibodies in succession as we previously reported [11]. DAPI was used to stain the nucleus. Photographs were taken at random fields, and results were from experiments in triplicate.

### 2.8. Real-Time PCR

SW620 cells were treated with various reagents at different concentrations for 24 h. RNA isolation and real-time PCR were performed according to the procedures we previously described [11]. Briefly, the total RNA was isolated using TRIzol reagent (Invitrogen, Carlsbad, CA, USA), and the first-strand cDNA was synthesized with 1 *μ*g of total RNA using a PrimeScript RT reagent kit (TakaraBio, Tokyo, Japan). The real-time PCR was conducted using IQTM SYBR Green supermix and the iQ5 real-time detection system (Bio-Rad Laboratories, Hercules, CA, USA). The reaction mixtures contained 7.5 *μ*l SYBR Green I dye master mix (Quanta), 2 pM forward primers, and 2 pM reverse primers. Thermocycle conditions included initial denaturation at 50°C and 95°C (10 min each), 40 cycles at 95°C (15 s) and 60°C (1 min). Glyceraldehyde phosphate dehydrogenase (GAPDH) was used as the invariant control. Relative mRNA levels were determined by the 2^−ΔΔCT^ method. Sequences of primers were listed in Table 1. Results were from experiments in triplicate.

### 2.9. Western Blot Analyses

SW620 cells were treated with various reagents at different concentrations for 24 h. Protein isolation and detection were performed according to the methods as we previously reported [11]. Briefly, the whole-cell lysates were prepared using radioimmunoprecipitation analyses buffer supplemented with protease and/or phosphatase inhibitors. In some experiments, mitochondrial and cytosol proteins were extracted, respectively, using a cytosol protein extraction kit (Nanjing KeyGen Biotech Co., Ltd., Nanjing, China) for detecting the release of Cyt c from mitochondria. In another set of experiments, nuclear proteins were prepared using a Bioepitope Nuclear and Cytoplasmic Extraction Kit provided by Bioworld Technology Co., Ltd. (Saint Louis Park, MN, USA). The protein concentrations were determined using a BCA assay kit (Pierce, USA). Proteins (50 *μ*g/well) were separated by SDS-polyacrylamide gel, and then transferred to a PVDF membrane (Millipore, Burlington, MA, USA) followed by being blocked with 5% skim milk in Tris-buffered saline containing 0.1% Tween 20. Target proteins were monitored by their primary antibodies, and subsequently by horseradish peroxidase-conjugated secondary antibodies. Protein bands were visualized using chemiluminescence reagent (Millipore, Burlington, MA, USA). Equivalent loading was confirmed using the antibodies against GAPDH, or COX IV, or lamin B1. The abundance of target proteins was densitometrically detected using Quantity Ones 4.4.1 (Bio-Rad Laboratories, Berkeley, CA, USA). The variation in the density of bands was presented as fold changes after normalized to GAPDH, or COX IV, or lamin B1, or the total proteins.

### 2.10. Statistical Analyses

Data were expressed as mean ± SD, and analyzed using SPSS16.0 software. The significance of difference was determined by one-way ANOVA with the* post hoc* Dunnett's test, and values of* P*<0.05 were set as statistically significant.

## 3. Results

### 3.1. *α*-Hederin Arrests Cell Cycle at G2/M Checkpoint in IL-6-Stimulated SW620 Cells

**Figure 1:**
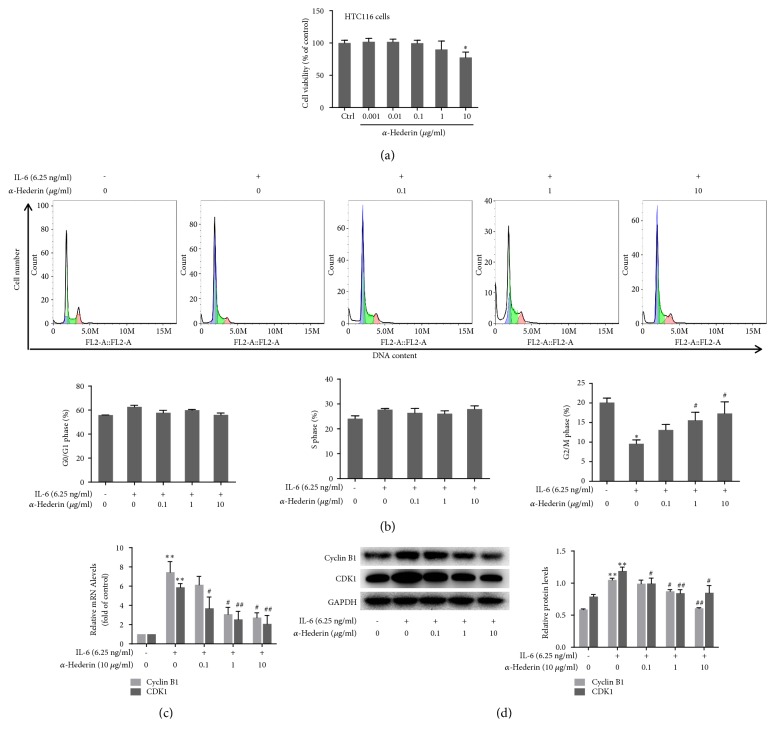
*α*-Hederin arrests cell cycle at G2/M checkpoint in IL-6-stimulated SW620 cells. SW620 cells or HCT116 cells were treated with vehicle, IL-6, and/or *α*-hederin at indicated concentrations for 24 h. (a) CCK-8 assay for evaluating cell viability. Cell viability was expressed as percentage of control. Significance: *∗P*<0.05 versus control. (b) Cell cycle analysis by flow cytometry. Percentages of cell cycle distribution at the G0/G1 phase, S phase, and G2/M phase were quantified, respectively. Significance: *∗P*<0.05 versus control, ^#^*P*<0.05 versus IL-6. (c) Real-time PCR analyses of mRNA expression of cyclin B1 and CDK1. Significance: *∗∗P*<0.01 versus control, ^#^*P*<0.05 versus IL-6, ^##^*P*<0.01 versus IL-6. (d) Western blot analyses of protein expression of cyclin B1 and CDK1 with quantification. Significance: *∗∗P*<0.01 versus control, ^#^*P*<0.05 versus IL-6, ^##^*P*<0.01 versus IL-6.

We initially examined the effects of *α*-hederin on the viability of colon cancer cells. Our previous studies have shown that *α*-hederin reduced the viability of SW620 cells at relatively low concentrations in a dose-dependent manner, and *α*-hederin at the low concentration of 1 *μ*g/ml produced a significant inhibitory effect [11]. Herein, *α*-hederin only at the high concentration of 10 *μ*g/ml significantly reduced the viability of HCT116 cells, suggesting that SW620 cells were more sensitive to *α*-hederin treatment (Figure 1(a)). Therefore, we used SW620 cells treated with IL-6 at 6.25 ng/ml for subsequent experiments. This concentration of IL-6 was determined by our previous studies [11]. Reduced cell viability can be caused by cell cycle arrest. We used flow cytometry to analyze the cell cycle, and found that *α*-hederin increased the number of cells at the G2/M phase, indicating that the cell cycle at the G2/M checkpoint was arrested (Figure 1(b)). Cyclins and cyclin-dependent kinases (CDKs) are known to control cell cycle, and, for the G2/M phase transition, cyclin B1 and CDK1 are critically involved [12]. We thus assumed that these two molecules might be affected by *α*-hederin. Real-time PCR analyses showed that IL-6 significantly increased the mRNA expression of cyclin B1 and CDK1, but *α*-hederin reduced their mRNA expression concentration dependently in IL-6-stimulated SW620 cells (Figure 1(c)). Western blot analyses consistently demonstrated that the protein levels of these molecules were also significantly elevated by IL-6, but were decreased by *α*-hederin in a concentration-dependent manner (Figure 1(d)). Collectively, these results suggested that *α*-hederin reduced viability and arrested cell cycle associated with downregulation of cyclin B1 and CDK2 in colon cancer cells.

### 3.2. *α*-Hederin Induces Mitochondrial Apoptosis in IL-6-Stimulated SW620 Cells

**Figure 2:**
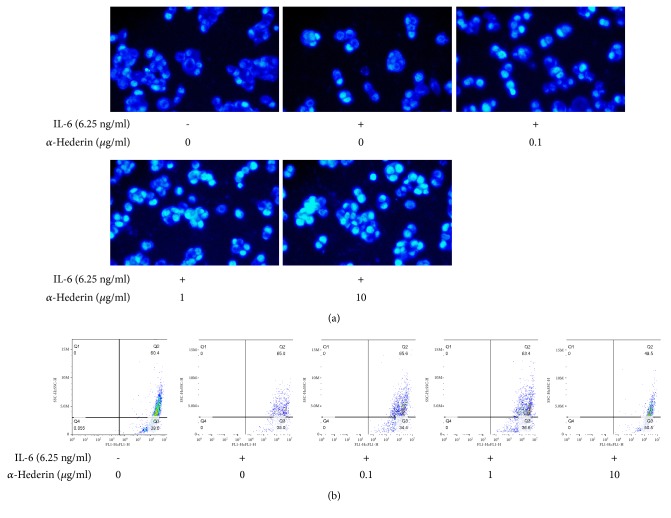
*α*-Hederin induces mitochondrial apoptosis in IL-6-stimulated SW620 cells. SW620 cells were treated with vehicle, IL-6, and/or *α*-hederin at indicated concentrations for 24 h. (a) Hoechst 33258 fluorescence staining. Morphologic changes of apoptotic cells were visualized under a fluorescence microscope (200 x magnification). (b) Flow cytometric analyses of MMP.

**Figure 3:**
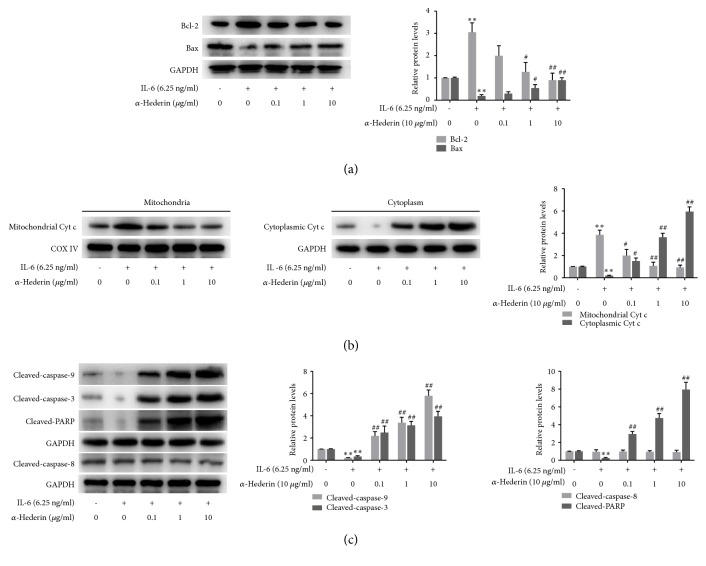
*α*-Hederin regulates mitochondrial apoptosis-related proteins in IL-6-stimulated SW620 cells. SW620 cells were treated with vehicle, IL-6, and/or *α*-hederin at indicated concentrations for 24 h. (a) Western blot analyses of protein expression of Bcl-2 and Bax with quantification. Significance: *∗∗P*<0.01 versus control, ^#^*P*<0.05 versus IL-6, ^##^*P*<0.01 versus IL-6. (b) Western blot analysis of mitochondrial and cytoplasm abundance of cytochrome c with quantification. Significance: *∗P*<0.05 versus control, *∗∗P*<0.01 versus control, ^#^*P*<0.05 versus IL-6, ^##^*P*<0.01 versus IL-6. (c) Western blot analysis of protein abundance of cleaved-caspase-9, cleaved-caspase-3, cleaved-caspase-8, and cleaved-PARP with quantification. Significance: *∗∗P*<0.01 versus control, ^#^*P*<0.05 versus IL-6, ^##^*P*<0.01 versus IL-6.

We next explored whether apoptosis was involved in *α*-hederin's inhibitory effects on colon cancer cells. Hoechst staining indicated DNA condensation and fragmentation with brilliant blue staining in IL-6-treated SW620 cells in the presence of *α*-hederin (Figure 2(a)). Flow cytometry analyses showed that *α*-hederin concentration-dependently reduced MMP in IL-6-treated SW620 cells (Figure 2(b)). The subsequent Western blot data demonstrated that *α*-hederin concentration-dependently reduced the expression of the anti-apoptotic molecule Bcl-2 and increased the expression of the proapoptotic molecule Bax in IL-6-treated SW620 cells (Figure 3(a)). In addition, increased release of Cyt c from mitochondria into cytoplasm induced by *α*-hederin was also observed (Figure 3(b)). Furthermore, *α*-hederin concentration-dependently upregulated cleaved-caspase-9, cleaved-caspase-3, and cleaved-PARP, whereas cleaved-caspase-8 was not apparently affected (Figure 3(c)), indicating the involvement of extrinsic apoptosis. Taken together, these findings strongly indicated that *α*-hederin triggered mitochondrial apoptosis pathway associated with activation of caspase cascades in colon cancer cells.

### 3.3. Blockade of NF-*κ*B Signaling Is Required for *α*-Hederin Induction of Mitochondrial Apoptosis in IL-6-Stimulated SW620 Cells

**Figure 4:**
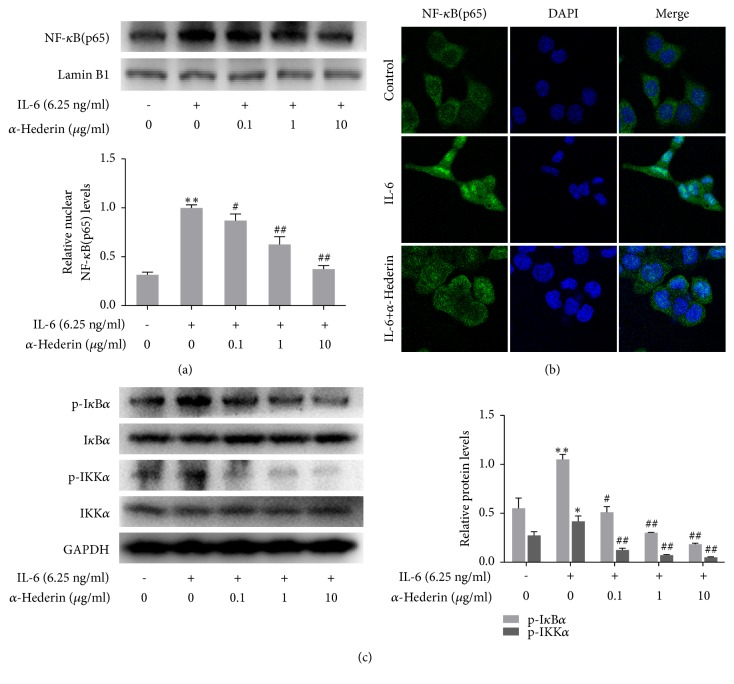
*α*-Hederin inhibits NF-*κ*B signaling in IL-6-stimulated SW620 cells. SW620 cells were treated with vehicle, IL-6, and/or *α*-hederin at indicated concentrations for 24 h. (a) Western blot analysis of nuclear abundance of NF-*κ*B with quantification. Significance: *∗∗P*<0.01 versus control, ^#^*P*<0.05 versus IL-6, ^##^*P*<0.01 versus IL-6. (b) Immunofluorescence analyses of the nuclear translocation NF-*κ*B in SW620 cells treated with *α*-hederin at 10 *μ*g/ml and/or IL-6 at 6.25 ng/ml (200 x magnification). (c) Western blot analysis of protein abundance of p-I*κ*B*α*, I*κ*B*α*, p-IKK*α*, and IKK*α* with quantification. Significance: *∗P*<0.05 versus control, *∗∗P*<0.01 versus control, ^#^*P*<0.05 versus IL-6, ^##^*P*<0.01 versus IL-6.

**Figure 5:**
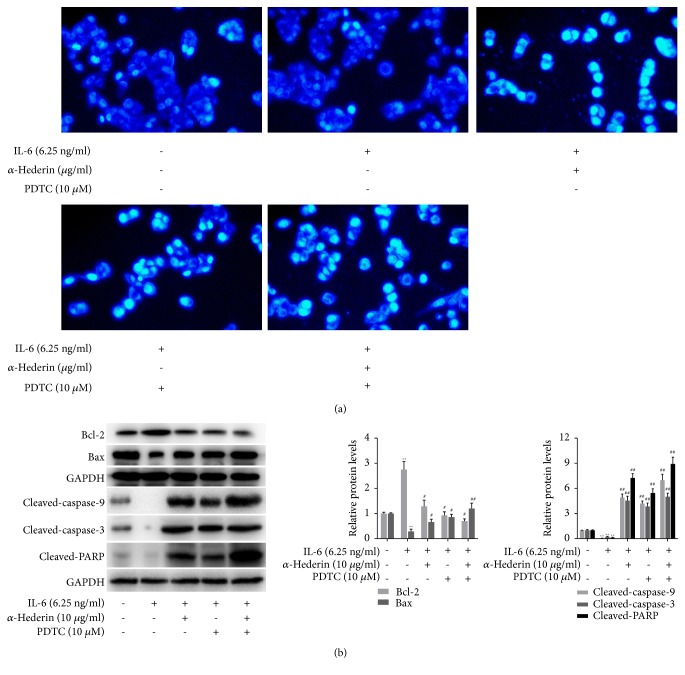
Blockade of NF-*κ*B signaling is required for *α*-hederin induction of mitochondrial apoptosis in IL-6-stimulated SW620 cells. SW620 cells were treated with vehicle, IL-6, and/or *α*-hederin or PDTC at indicated concentrations for 24 h. (a) Hoechst 33258 fluorescence staining. Morphologic changes of apoptotic cells were visualized under a fluorescence microscope (200 x magnification). (b) Western blot analysis of protein abundance of cleaved-caspase-9, cleaved-caspase-3, and cleaved-PARP with quantification. Significance: *∗∗P*<0.01 versus control, ^#^*P*<0.05 versus IL-6, ^##^*P*<0.01 versus IL-6.

**Figure 6:**
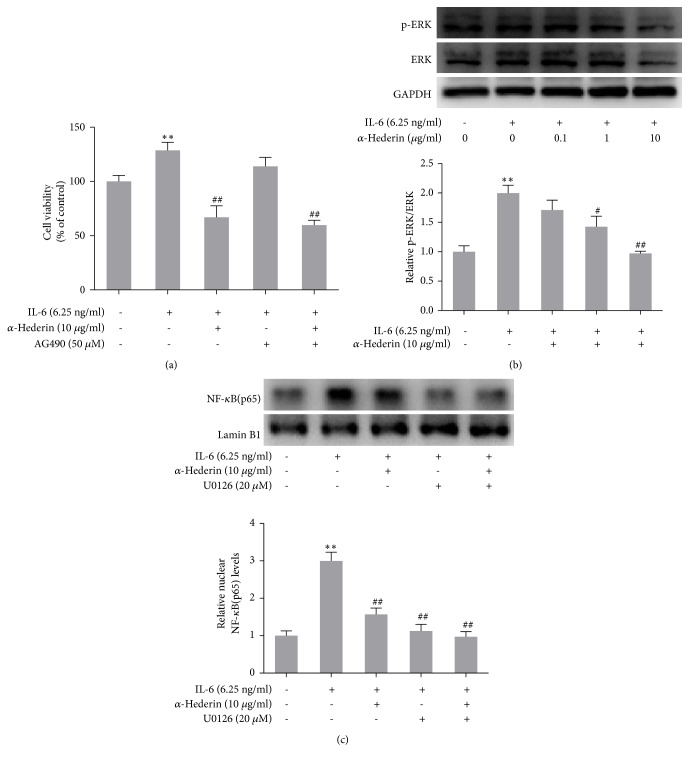
Inhibition of ERK phosphorylation is involved in *α*-Hederin reduction of NF-*κ*B nuclear translocation in IL-6 stimulated SW620 cells. SW620 cells were treated with vehicle, IL-6, and/or *α*-hederin, or AG490, or U0126 at indicated concentrations for 24 h. (a) CCK-8 assay for evaluating cell viability. Cell viability was expressed as percentage of control. Significance: *∗∗P*<0.01 versus control, ^##^*P*<0.01 versus IL-6. (b) Western blot analysis of ERK phosphorylation with quantification. Significance: *∗∗P*<0.01 versus control, ^#^*P*<0.05 versus IL-6, ^##^*P*<0.01 versus IL-6. (c) Western blot analysis of nuclear abundance of NF-*κ*B with quantification. Significance: *∗∗P*<0.01 versus control, ^##^*P*<0.01 versus IL-6.

We investigated the involvement of NF-*κ*B signaling in order to define the mechanisms underlying *α*-hederin-induced colon cell apoptosis. Western blot analyses of nuclear lysate showed that IL-6 increased the nuclear abundance of NF-*κ*B, but *α*-hederin concentration dependently reduced NF-*κ*B nuclear abundance (Figure 4(a)). Immunofluorescence staining demonstrated consistent results, showing that *α*-hederin at 10 *μ*g/ml obviously abolished IL-6-induced NF-*κ*B nuclear translocation in SW620 cells (Figure 4(b)). Moreover, IL-6 apparently increased the phosphorylation of I*κ*B*α* (the deactivated form of this molecule), the inhibitor of NF-*κ*B; but *α*-hederin concentration-dependently decreased I*κ*B*α* phosphorylation (Figure 4(c)). IL-6 also apparently increased the phosphorylation of IKK*α* (the activated form of this molecule), the inhibitor of IKK*α*; but *α*-hederin concentration dependently decreased I*κ*B*α* phosphorylation (Figure 4(c)). These results clearly demonstrated that *α*-hederin blocked NF-*κ*B signaling, which could be associated with the induction of apoptosis in colon cancer cells. To validate this association, NF-*κ*B selective inhibitor PDTC was used to treat SW620 cells alone or combined with *α*-hederin. Hoechst staining results showed that PDTC induced apoptosis in SW620 cells and its combination with *α*-hederin produced more potent effects (Figure 5(a)). Furthermore, PDTC, similar to *α*-hederin, downregulated the Bcl-2 expression and upregulated the abundance of Bax, cleaved-caspase-9, cleaved-caspase-3, and cleaved-PARP in IL-6-treated SW620 cells, and notably, combination of PDTC and *α*-hederin resulted in more potent regulatory effects on the protein expression of these molecules (Figure 5(b)). On the other hand, our recent studies suggested that *α*-hederin inhibited epithelial-to-mesenchymal transition (EMT) by interrupting JAK2/STAT3 signaling in SW620 cells [11]. Here, our additional assays showed that JAK/STAT3 signaling inhibitor AG490 at 50 *μ*M, unlike *α*-hederin, failed to repress the viability of SW620 cells (Figure 6(a)), suggesting that the JAK2/STAT3 signaling might specifically regulate the EMT of colon cancer cells. Moreover, the ERK signaling plays a key role in cancer cell fate decision [13]. We here observed that *α*-hederin reduced IL-6-indcued ERK phosphorylation in SW620 cells (Figure 6(b)). ERK inhibitor U0126 at 20 *μ*M inhibited NF-*κ*B nuclear accumulation in SW620 cells, and combination of U0126 and *α*-hederin produced more potent inhibitory effects (Figure 6(c)), suggesting that inhibition of ERK signaling could be an upstream molecular event for *α*-hederin-induced apoptosis in SW620 cells. Taken together, these results suggested that *α*-hederin blockade of NF-*κ*B signaling was involved in induction of mitochondrial and caspase-dependent apoptosis in colon cancer cells.

## 4. Discussion

Increasing evidence suggests *α*-hederin as a good candidate for cancer chemotherapy. Herein, we treated colon cancer cells with IL-6 to mimic the paracrine inflammatory microenvironment of tumor cells. We found that *α*-hederin significantly reduced cell viability and induced apoptosis in a concentration-dependent manner in colon cancer cells. Our study demonstrated that *α*-hederin caused G2/M arrest in SW620 cells, resulting in decreased cell viability. Cell proliferation is controlled by cell cycle progression, which is a highly regulated process [14]. The cell cycle is constituted by four non-overlapping phases in sequence, namely, the G1, S, G2, and M phases. Each phase contains a checkpoint that can arrest cell cycle arrest and initiate repair mechanisms [14]. Normal cells commonly use the G1 checkpoint to repair DNA damage. Tumor cells, however, are more dependent on the G2 checkpoint for protecting against DNA damage [15]. These discoveries highlight the G2 checkpoint as a selective target for treatment of cancer. In addition, cell cycle is mediated by a highly conserved protein kinase family. Cyclins can activate CDKs through forming complexes with CDKs, among which the cyclin B1/CDK1 complex is critically important for the G2 to M phase transition [16]. In the present study, flow cytometric analyses showed that *α*-hederin induced G2/M phase cell cycle arrest in colon cancer cells, and G2/M phase accumulation peaked at 24 h of treatment, suggesting the occurrence of sequential events of cell cycle arrest. Furthermore, G2/M phase arrest is known to be mediated by reduced formation of cyclin B1/CDK1 complex during cell cycle progression [17]. In current study, we found that *α*-hederin arrested SW620 cells in G2/M phase through downregulating the expression of cyclin B1 and CDK1 at both transcriptional and protein levels. This could result in reduced abundance of cyclin B1/CDK1 complex within cells. Our findings were consistent with the established molecular recognition and strongly suggested that *α*-hederin could be developed as a selective agent for colon cancer treatment.

To elucidate the underlying mechanism, we examined *α*-hederin's effects on apoptosis in colon cancer cells. Cell cycle arrest induced by drugs can cause inefficient repair, leading to apoptosis if the damage is unrepairable [4]. Mitochondria are the major organelles involved in apoptosis signaling. Mitochondrial apoptosis pathway can be initiated by intracellular stimuli and mediated by the Bcl-2 family proteins, which function as sensors to integrate the survival and death signals. The ratio of Bcl-2/Bax is a pivotal determinant, and reduced Bcl-2/Bax ratio can result in mitochondrial outer membrane permeabilization and Cyt c release, and finally activate caspase-9 and caspase-3, culminating in cellular fragmentation [18, 19]. Here, our data demonstrated that *α*-hederin led to decreased ratio of Bcl-2/Bax and disrupted MMP accompanied by increased release of Cyt c into cytoplasm, suggesting the initiation of mitochondrial-mediated apoptosis. In addition, caspase-9, caspase-3, and PARP-1 were all activated, indicating caspase-associated apoptosis induced by *α*-hederin. Interestingly, the extrinsic apoptosis pathway might not be involved, because caspase-8 was not markedly activated. Taken together, these findings suggested that *α*-hederin selectively stimulated colon cancer cells to undergo intrinsic apoptosis dependent on caspase activation.

NF-*κ*B can promote cell survival and proliferation. Increased NF-*κ*B activity is positively associated with many types of cancers [20]. Thus we investigated the expression and phosphorylation of some key components in NF-*κ*B pathway to further reveal the mechanisms underlying *α*-hederin-induced apoptosis. We demonstrated that *α*-hederin inhibited IL-6-induced nuclear translocation of NF-*κ*B. These effects could prevent NF-*κ*B to transcriptionally activate the genes involved in cell proliferation. Disruption of NF-*κ*B pathway by *α*-hederin contributed to its inhibitory effects on cell cycle progression and apoptosis induction in colon cancer cells. These molecular insights could make *α*-hederin a more valuable candidate for cancer chemotherapy, given that invasion and metastasis of tumor cell are closely related to inflammation, while NF-*κ*B is exactly a pivotal player in inflammation-induced tumor progression and metastasis [21]. Inhibition of NF-*κ*B could lead to innovative approach for the treatment of cancer [20]. Our investigation demonstrated that *α*-hederin disrupted NF-*κ*B signaling in colon cancer cells, contributing to its induction of mitochondrial apoptosis. Furthermore, we preliminary explored the upstream molecule event of *α*-hederin effects. We ruled out the role of JAK2/STAT3 signaling, because pharmacological inhibition of this pathway had little effect on the viability of SW620 cells. We turned to the ERK signaling, which has a broad array of cellular functions including proliferation, survival, apoptosis, motility, transcription, metabolism and differentiation [22]. We observed that *α*-hederin inhibited ERK signaling contributing to the disruption of NF-*κ*B signaling and presumably to the induction of apoptosis in colon cancer cells. These findings were consistent with a number of previous studies where inhibition of ERK activity led to or associated with apoptosis of colon cancer cells [23–28]. However, there were also contrast results showing that activation of ERK induced apoptosis in colon cancer cells [29, 30]. To account for the discrepancies, we will deeply investigate the distinct mechanisms underlying *α*-hederin inhibition of ERK signaling implicated in regulation of colon cancer cell fate.

In conclusion, *α*-hederin reduced viability, arrested cell cycle at the G2/M checkpoint, and induced mitochondrial and caspase-dependent apoptosis in SW620 cells. These effects were associated with the blockade of NF-*κ*B signaling and inhibition of ERK. Our results strongly suggested *α*-hederin as a promising candidate for colon cancer therapy.

## Figures and Tables

**Table 1:** Primer sequences of genes for real-time PCR in this study.

Genes	Sequences	
Cyclin B1	Forward	5orwACGAAGGTCTGCGCGTGTT-3′
	Reverse	5eveCCGCTGGCCATGAACTACCT-3′

CDK1	Forward	5orwardGCAGACTTTGGACTAGCCAG-3′
	Reverse	5everseCGGTACCACAGGGTCA-3′

GAPDH	Forward	5′-TGACAACAGCCTCAAGAT-3′
	Reverse	5′-GAGTCCTTCCACGATACC-3′

## Data Availability

The data used to support the findings of this study are available from the corresponding author upon request.
